# Basket Trials in Oncology: A Trade-Off Between Complexity and Efficiency

**DOI:** 10.1200/JCO.2016.69.9751

**Published:** 2016-11-28

**Authors:** Kristen M. Cunanan, Mithat Gonen, Ronglai Shen, David M. Hyman, Gregory J. Riely, Colin B. Begg, Alexia Iasonos

**Affiliations:** Kristen M. Cunanan, Mithat Gonen, Ronglai Shen, David M. Hyman, Gregory J. Riely, Colin B. Begg, and Alexia Iasonos, Memorial Sloan Kettering Cancer Center, New York, NY.

The current oncology drug development landscape is dominated by efforts to create therapies that are mechanistically designed to improve outcomes for patients with cancers that harbor specific molecular aberrations, which often occur across a variety of tumor types. In the evaluation of targeted therapies, basket trials have emerged as an approach to test the hypothesis that targeted therapies may be effective independent of tumor histology, as long as the molecular target is present.^1^ However, the term basket has been applied broadly, and there is little uniformity in the design or goals of these trials. Furthermore, the scientific goals frequently are not specified with the precision conventionally used for clinical trials, leading to some difficulties in design and interpretation. For instance, many investigative teams use the popular Simon two-stage design, independently in each basket, thus effectively treating the trial overall as a series of independent phase II clinical trials. However, the actual goals are typically more complex than those of simple phase II clinical trials of new agents. In this commentary, we present an overview of the various trials described as basket trials, clarify the distinctive goals that basket trials seek to address, discuss the inherent hidden complexities, and offer general recommendations regarding their design.

Several approaches to evaluating targeted therapies in multiple tumor types have been described as basket trials (Fig 1). The first prototype in Figure 1 is the basket trial of vemurafenib.^2^ Vemurafenib is an oral inhibitor of *BRAF* that has greater selectivity for the *BRAF*^V600^ mutant form of the kinase than for wild-type *BRAF*, which had been previously approved for patients with *BRAF*^V600E^ mutation–positive metastatic melanoma. Vemurafenib was targeted at a single variant in a variety of cancers with different primary disease sites and histologies, thereby defining disease-specific baskets. The second prototype in Figure 1 is the CREATE trial,^3^ which evaluated the use of the anaplastic lymphoma kinase and/or mesenchymal-epithelial transition factor inhibitor crizotinib. Here, although again there was a single agent under investigation, the drug inhibits multiple oncokinases including c-Met and anaplastic lymphoma kinase. Thus the baskets reflect a combination of diseases and targets. The last prototype in Figure 1 is the CUSTOM trial.^4^ In this trial, investigators planned to enroll patients with one of three diseases and allocate them to one of five targeted therapies, resulting in 15 disease-drug-mutation–specific baskets. Thus, this study tests the efficacy of a variety of drugs in a variety of targets and disease sites. We note that more complex trials than those presented in Figure 1, such as the NCI-MATCH,^5^ Genentech MyPathway,^6^ Novartis Signature,^7^ and American Society of Clinical Oncology’s Targeted Agent and Profiling Utilization Registry Study (TAPUR)^8^ basket trials, fall into this general framework. These ongoing studies define drug-mutation–specific baskets because their aim is to determine the efficacy of drugs that target certain pathways, typically with postmarketed drugs in nonindicated solid tumor types. The NCI-MATCH^5^ trial is even more complex in that genomic screening is incorporated into the therapeutic study itself and treatment assignment is determined by a matching algorithm that uses predefined levels of evidence of the gene variants. The trial aims to assess the activity of multiple drugs used in mutation-specific baskets (mutations, amplifications, or translocations), regardless of tumor origin, using a single stage design for each biomarker-defined subgroup (ie, mutation-specific basket).^5^ As the clinical setting becomes more complex for a study, the terms basket trial and umbrella trial begin to overlap. For example, the NCI-MATCH trial has been referred to as both an umbrella trial as a result of the multiple drugs under evaluation^5^ and as a basket trial because of the multiple disease populations for screening.^1,9^ Moreover, many basket studies evaluate multiple genomic variants in a given gene, which further complicates the clinical setting, and these variants may individually influence the likelihood of response to therapy.

**Fig 1.**
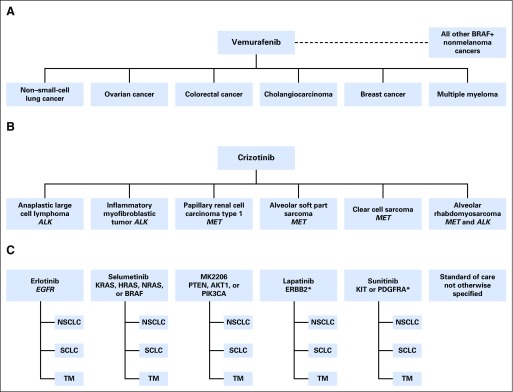
Three published basket trials. (A) Disease-specific baskets.^2^ (B) Disease-mutation–specific baskets (CREATE).^3^ (C) Disease-drug-mutation–specific baskets (CUSTOM).^4^ *Mutations or amplifications. *ALK*, anaplastic lymphoma kinase; *EGFR*, epidermal growth factor receptor; *MET*, mesenchymal-epithelial transition factor; NSCLC, non–small-cell lung cancer; SCLC, small-cell lung cancer; TM, thymic malignancy.

All the trials in Figure 1 were constructed as a series of independent phase II trials, using a conventional two-stage design, such as the Simon design,^10^ within each basket, individually controlling type I error at a nominal level. However, the design aspects and performance characteristics of these trials are not well understood, and the nature of the scientific goals is more complex than those of traditional disease-specific studies.^1^ Most clinical trials are constructed to address a single primary objective. Although secondary objectives may be articulated in the protocol, the chosen study design may not be ideal for addressing all of them, although in conventional clinical trials, the overall design is frequently suitable for addressing typical secondary goals. In the context of a basket trial, the ideal design options are not necessarily well aligned for the numerous questions being asked. Thus, careful consideration is needed to identify which question is paramount and to design the study accordingly. To focus our discussion, we limit attention to the seemingly most straightforward setting in which there is one target mutation and one drug targeting that mutation, evaluated in several disease types. In this setting, the questions the investigators seek to address may be one or more of the following: Does the drug have any efficacy at all? Does efficacy differ by disease site? If so, in which disease sites does the drug work?

Consider the first question: Does the drug have any efficacy at all? This is the essential question for determining whether to pursue additional studies that would lead to regulatory approval for marketing the drug. A basket study that implements multiple independent two-stage designs (one per basket) has a much higher false-positive rate than a typical phase II study (ie, there is a much higher chance that the drug will be declared effective in at least one basket when in fact the drug is truly ineffective). For example, if there are five baskets and each has a 5% false-positive rate, the chance that an ineffective drug will be declared effective in one or more baskets is approximately 23%; increasing the number of baskets exacerbates this false-positive rate (for 10 baskets, approximately 40%). In short, the common practice of using a basket trial design that treats each cohort as an independent trial inevitably leads to a higher overall false-positive rate. Investigators can control the overall false-positive rate by adjusting the sample size or decision rules so that there is a lower false-positive rate within each basket. At a minimum, it is important to report the overall false-positive rate in a basket trial.

If the primary goal of the study is to determine the effect of the drug separately in each basket, a design structured to evaluate drug efficacy in each cancer site independently, such as a series of independent Simon designs, is an appropriate candidate. However, this strategy fails to recognize the potential inherent connectedness of the efficacy results in the different baskets and our expectations regarding this. As positive results emerge in a new cancer site, they increase our expectation of positive results in other sites, as has been observed recently in studies targeting kinase fusions.^11^ At the outset of the trial, we expect the efficacy results to be correlated. Evidence of such correlation can be harnessed productively in such trials, and there are two distinct ways of taking advantage of this. The first is the concept of aggregation.^12,13^ If, as the trial progresses, we observe good evidence of similar efficacy in a subset of the baskets, then aggregation of these baskets on the basis of an interim analysis can allow us to reach conclusions for this subset of baskets more quickly (ie, with fewer patients). The second concept is the use of statistical modeling. In this framework, the similarity of the results between baskets can be factored into a statistical model.^14,15^ This strategy allows the information from responses in different baskets to be shared, improving the efficiency of the statistical design and, by implication, permitting the study to reach conclusions with fewer patients.

The use of these concepts has the potential to greatly improve the design of basket trials, but additional complexities must be recognized. The concept of statistical power becomes more complex in the basket setting. Power is the probability that the drug will be shown to be effective if it is truly effective, and in phase II trials, the calculation involves specifying the hypothesized true effectiveness of the drug. In basket trials, we must consider different configurations of effectiveness. For example, the drug may truly work only in one basket. Alternatively, it may actually work in two of the baskets, or in three or more. Each of these configurations can lead to markedly different statistical properties, and thus, the ideal study design is different, depending on which of these scenarios is true.

Recent research on a design and analysis strategy that permits aggregation of baskets with similar efficacy results, as determined by an interim assessment of heterogeneity, has shown large potential gains in efficiency in determining if the drug works overall.^12^ Efficiency is represented by the need for fewer patients, and it can be shown that this is improved, especially when the drug truly works across most of the baskets under investigation, with a modest cost with respect to power if the drug is effective in only one of the baskets. We display some results from this research that show considerable reductions in both the expected (ie, average) sample sizes and trial durations in settings in which the drug is truly effective in the preponderance of the baskets (Appendix Fig A1, online only). However, this strategy has limitations if the primary goal is to classify the effectiveness of the drug in each individual basket, because this goal requires adequate accrual to each basket. These results demonstrate that it is inevitable that the choice of design must involve compromise, balancing the ideal properties when the drug truly works in different numbers of baskets. In this context, interim analyses can be highly informative and can be constructed to make interim decisions that use the accumulating evidence more efficiently than simply following the decision rules embedded in independent Simon two-stage designs.

In summary, the advent of targeted therapy has led to the introduction of a new class of clinical trials, and the term basket trial has come to be used to cover many different trial designs in this class. These different types of clinical trials share the characteristics that one or more targeted therapies are being tested and that the drugs are being investigated in distinct disease subtypes or sites. The scientific goals of these trials are typically more complex and frequently not specified with the precision conventionally used for clinical trials. Most investigators view a basket trial as a series of independent phase II clinical trials. In fact, the simplest type of basket trial, the evaluation of a single drug targeting a single mutation in multiple disease sites, presents a much more complex framework than a conventional evaluation of a single drug in a single disease. We believe that creative investigation into design options offers the potential to meet the study goals faster, with fewer patients. Such investigation must recognize the fact that most basket trials typically aim to answer multiple questions simultaneously. Most importantly, in this period of transition to precision medicine, our clinical research tools must maintain the scientific rigor embedded in the traditional clinical trials paradigm, in which hypotheses are specified precisely and the clinical trial is designed to address these hypotheses.

## Figures and Tables

**Fig A1.**
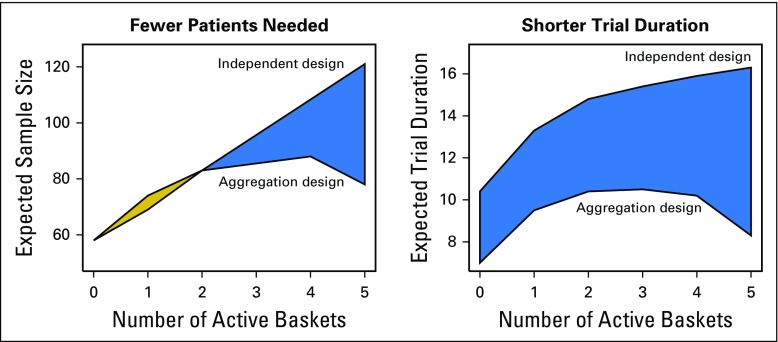
Gains and losses in sample size and trial duration (months) for two designs. The blue highlighted area shows gains when using an aggregation design; the gold highlighted area shows losses when using independent parallel phase II studies.

## Appendix

### Clinical Setting

Investigate five disease-specific baskets, assuming a 15% null response rate and a 45% desirable response rate. Accrual assumed to be two patients per month.

### Independent Design

Implements independent, parallel, two-stage Simon designs with 1% type 1 error rate and 20% type 2 error rate (ie, 80% power) within each basket. With these specifications, the family-wise error rate is controlled at 5%.

### Aggregation Design

Implements an interim heterogeneity assessment to potentially aggregate baskets after first stage. This design is calibrated to control the family-wise error rate to 5% and to achieve at least 80% marginal power when the drug truly works in two or more (of the five) baskets, where marginal power is defined as the probability of correctly identifying that the drug works in an individual basket.

### Interpretation of Results

In Figure A1, the blue shaded areas highlight the improved efficiency of the aggregation design in most configurations, in terms of expected (ie, average) sample size (left panel) and expected trial duration (right panel). The aggregation design is modestly inefficient with respect to sample size when the drug truly works in only one basket (gold shading).
